# Supplementary material for: Are carbon emissions trading and green financial instruments synergistic? -Comprehensive quantitative research based on content analysis
Source: PLoS One. 2024 Mar 7;19(3):e0298601. doi: 10.1371/journal.pone.0298601 (PMC10919601; doi:10.1371/journal.pone.0298601)
Supplement: S1 Data — (ZIP) [file pone.0298601.s001.zip › Data/地级市Z_F工作报告.zip!/╔╜╬≈/╦╖╓▌╩╨/└┤╫╘╣1⁄2╓┌║┼í╛┬φ┐╦╩2╛▌═°í┐_2008.docx]

各位代表：

　　现在，我代表市人民政府向大会作工作报告，请予审议，并请市政协各位委员和其他列席人员提出意见。

　　一、2007年政府工作回顾

　　2007年，在中共朔州市委的正确领导和市人大、市政协的有力监督支持下，我们团结带领全市人民，坚持以邓小平理论和“三个代表”重要思想为指导，认真贯彻党的十七大精神，全面落实科学发展观，紧紧围绕“两个翻番”和“两大基地”建设目标，团结拼搏，扎实苦干，全市各项工作都呈现出前所未有的大好势头，成为建市以来经济发展最快、社会效益最好、人民得到实惠最多的一年。

　　国民经济强劲增长，综合经济实力跨上新台阶。地区生产总值达到330.2亿元，比上年增长15.8%；人均达到21696元，增长15.1%。财政总收入达到80亿元，增长53.1%；一般预算收入达到30.2亿元，增长61.3%，两项指标增幅均居全省第一。规模以上工业增加值增长26%，实现利税增长28.1%。投资、消费、出口等拉动经济增长的三大要素，增幅均居全省首位。固定资产投资达到153亿元，增长60.8%；社会消费品零售总额增长24.5%；海关进出口总额增长2.17倍。

　　招商引资再创佳绩，经济结构调整步伐加快。全市共签订招商引资项目122个，履约104个，开工建设34个，到位资金130亿元，签约项目数、合同履约率和资金到位率均居全省前茅。新开工重点调产项目178个，在建项目总数达到290个，其中111个基本建成或投产运行。煤炭产业素质进一步提高，地方煤矿减少到136座，30万吨以上煤矿占到矿井总数的三分之二，全市煤炭生产能力达到1.17亿吨，全省第一。单独保留的91座矿井全部铺开了采煤方法改革。电力工业发展势头看好，8家电厂列入国家电力发展规划，平朔和格瑞特两家已开工建设。农畜产品加工、陶瓷、化工、新材料等新兴工业和服务业快速成长，全市非煤非电项目占到在建项目总数的75%，一批项目已正式投产。

　　新农村建设稳步推进，农业农村经济快速发展。种植业结构进一步优化。优质玉米、马铃薯、小杂粮、蔬菜、甜菜、葫麻六大基地面积占到农作物总播面积的85%以上，具有区域特色的种植结构体系初步形成。全市粮食总产达到7.3亿公斤，连续三年刷新历史纪录。畜牧业抗住了严重市场冲击，继续保持稳定发展势头。全市奶牛存栏量13.86万头，增长14.4%；生猪和羊存栏量分别增长11.9%和1.7%。新农村建设取得阶段性成果。200个试点村和重点推进村的“五个一工程”建设和“四化四改”工作均圆满完成省定任务。19个“两区”开发项目，其中4个已竣工投产，14个正在建设。国土资源保护工作取得明显成效。省委、省政府表彰的五项农村重点工作，我市项项获奖。

　　基础设施和生态文明建设力度空前，发展硬环境进一步改善。城市建设大规模展开。市区共铺开重点基础设施建设工程20项，是建市以来项目最多、投入最大的一年。七里河大桥扩建、学苑小区过街天桥、七里河公园景观改造等8项工程已经竣工，文化艺术中心、大运路市区段改造、民福街立交桥等9项工程顺利开工。城市绿化、净化、亮化工作成效明显，建成区绿化覆盖率达到42.2%，人均公共绿地面积达到9.8平方米，荣获省级园林城市称号。县区旧城改造规模不断扩大。公路建设取得新进展。新增公路通车里程1097公里，所有建制村实现通公路，83.6%的建制村实现了通油(水泥)路，92%的建制村实现了通班车。生态环境保护力度加大。坚决淘汰落后产能，加快了燃煤电厂脱硫除尘工程建设，取缔了市区周边所有土小白灰窑，拆除了一批燃煤锅炉，全市二氧化硫排放量下降5.2%，化学需氧量下降3.7%，均超额完成省定目标；市区空气质量二级以上天数达到300天，比上年增加16天。植树造林投资2亿多元，完成造林27万亩，植树1800多万株，得到省委、省政府的充分肯定。

　　各项社会事业加速发展，“和谐朔州”建设提高到一个新水平。教育事业全面发展。完成了100所农村寄宿制学校建设，为55所学校配置了暖气。高考二本以上达线人数再创历史最好水平。市职业技术学院竣工招生，师范专科学校获得批准。科技研发取得突破性进展。共取得专利52项，其中国际专利2项。平朔高新技术研发中心利用粉煤灰提取二氧化硅和氧化铝技术达到国际领先水平。医疗卫生事业进一步发展。完成了11所乡镇卫生院和15个社区卫生机构建设，县乡村三级医疗卫生机构达标率达到60%，新型农村合作医疗由3个县区扩大到5个县区，市中心医院、急救中心、疾控中心全部投入使用。人口计生工作受到省政府表彰。群众文化生活繁荣活跃，大秧歌剧《泥窑》参加了全国非物质文化稀有剧种展演，第九届广场文化艺术节暨第三届网络文化节成功举办。群众性体育活动丰富多彩。市区广播电视数字化改造基本完成。精神文明和民主法制建设进一步加强。社会治安综合治理和安全生产监管措施得到有效落实。国防教育不断深入开展。统计、地震、气象、档案史志、民族宗教、妇女儿童、老龄、残疾人等各项事业也都取得新成绩。

　　民生问题得到有效改善，人民生活水平进一步提高。解决了6640名农村贫困人口和低收入人口的温饱问题。新增城镇就业岗位2.5万个。企业养老、失业、医疗等七项社会保障制度和特困群众医疗救助、贫困家庭子女就学资助等十二项社会救助制度进一步完善，

　　企业离退休人员基本养老金标准连续三年得到提高，特别是在朔城区、平鲁区推行了65岁以上农村居民生活补贴制度，开创了全国先河。县区住房公积金制度全面启动，完成安康住房56.7万平方米，廉租住房1636平方米。城镇居民人均可支配收入达到11779元，增长19.6%，扭转了连续数年在全省中下游徘徊的局面；农民人均纯收入达到4146元，增长22%，增幅全省第一。

　　政府自身建设进一步加强，政府公信力、执行力和凝聚力进一步增强。市政府坚持依法行政，自觉接受市人大及其常委会的监督，积极加强与市政协及各民主党派、各人民团体的联系，办理人大代表意见建议和政协委员提案120余件，做到了件件有回音，事事有落实。积极推进了政务公开，我市被评为全国先进单位。加快了政府职能转变，建立健全了政府应急管理体系。全面落实党风廉政建设责任制，切实加强了对药品集中采购、土地出让、工程建设的监察与审计，严肃查处了一批侵害群众利益的违法违纪案件。大力加强机关效能建设，机关作风明显改进，效率显著提高。

　　总之，经过全市人民一年的共同努力，我市经济社会发展取得了巨大的成就。这里，我郑重向各位代表报告：市四届人大四次会议确定的各项年度奋斗目标，除物价指数外，其余均已圆满完成!年初承诺为群众办好的十六件实事，除了从神头电厂引热入市因方案调整没有实施外，其余十五件均已圆满完成!市四届人大三次会议确立的“十一五”地区生产总值和财政总收入“两个翻番”目标，经过连续两年的艰苦奋战，地区生产总值已完成“十一五”目标的90.9%，接近翻番；财政总收入已完成目标的107%，提前三年实现翻番!

　　各位代表，成绩确实来之不易。这些成绩的取得，是省委、省政府和市委正确领导的结果，是市人大、市政协及各民主党派、各人民团体鼎力支持的结果，是全市各级各部门和广大人民群众团结奋斗、不懈努力的结果。在此，我代表市人民政府，向为朔州改革开放和现代化建设作出重大贡献的全市人民致以崇高的敬意!向所有关心、支持和参与朔州改革发展大业的同志们、朋友们，表示衷心的感谢!

　　当然，我们也清醒地认识到，我市处于欠发达地区中的落后地位仍然没有改变，艰苦创业、负重加压，加快发展、后来居上仍然是一项长期而艰巨的任务。

　　目前，全市经济社会发展中还存在着许多不容忽视的矛盾和问题。经济总量仍然很小，支柱产业单一、发展方式粗放的问题仍很突出；农业综合生产能力尚未得到明显提高，农民持续增收难度加大；城市功能不全，社会事业发展相对滞后，就业、环境和安全生产压力与日俱增，构建和谐社会、维护治安稳定的任务十分繁重；社会保障水平仍然较低，不少城乡居民生活还比较困难；一些干部的思想作风、工作效率、服务质量还有待于进一步改进和提高，投资环境还有待于进一步改善。这些矛盾和问题，都需要我们认真对待，全力解决。我们一定要居安思危，以对党对人民高度负责的态度，自我加压、负重赶超、顽强拼搏、团结奋进，努力回报全市150万人民的信任与厚望!

　　二、2008年政府工作总的指导思想和主要目标任务

　　今年是全面贯彻落实党的十七大精神的第一年，是奥运之年和改革开放三十周年，也是我们实现市委四届三次全会提出的未来三年地区生产总值和财政总收入“两个再翻番”、建设塞外最宜居最宜发展城市宏伟目标的起始之年、关键之年，而明年又是建市二十周年。我们一定要自觉肩负起历史赋予我们的光荣而艰巨的使命，在市委的坚强领导下，勇挑重担，冲锋在前，团结带领全市人民，坚持把发展作为最大的政治、最硬的道理、最根本的任务，拿出大气魄，实施大动作，推动大发展，在新的历史起点和高度上，再铸朔州新辉煌!

　　今年政府工作总的指导思想是：认真贯彻落实党的十七大精神，高举中国特色社会主义伟大旗帜，以邓小平理论和“三个代表”重要思想为指导，全面落实科学发展观，按照市委四届三次全会部署，大力弘扬“豪爽大气、海纳百川、百折不挠、奋力赶超”的新朔州精神，紧紧围绕“两个再翻番”目标，牢牢抓住调整经济结构、转变发展方式这条主线，以新型工业化、特色城镇化、农业现代化为抓手，加快“两大基地”(新型能源工业、生态畜牧经济)建设和服务业发展，积极推进和谐社会建设和民主法制建设，着力改善民生，实现经济、政治、文化、社会和生态文明建设的全面进步，努力提高全市综合经济实力、可持续发展能力、和谐社会水平和人民生活质量，为把我市建设成为塞外最宜居最宜发展城市而奋斗!

　　今年，我市经济社会发展的指导性指标是：地区生产总值达到420亿元，增长12%；规模以上工业增加值达到230亿元，增长20%；全社会固定资产投资达到190亿元，增长24%；社会消费品零售总额达到94亿元，增长15.2%；外贸进出口总额完成7330万美元，增长12%；财政总收入达到96亿元，增长22%，力争100亿元；一般预算收入达到34.5亿元，增长19.4%；粮食总产达到7.3亿公斤。

　　约束性指标是：服务业增加值增幅高于地区生产总值增幅；研发投入占GDP的比例达到1%；万元地区生产总值综合能耗下降5.6%；二氧化硫和化学需氧量排放削减12.12%和9.23%以上；居民消费价格总水平涨幅控制在4.7%以内；城镇居民人均可支配收入达到13200元，增长12%；农民人均纯收入达到4480元，增长8%；城镇新增就业岗位1.64万个，转移农村剩余劳动力3万人，城镇登记失业率控制在3%以内；城市义务教育阶段免费教育全面实施；人口自然增长率控制在6‰以内。

　　完成上述目标任务，必须牢牢抓住发展这个第一要务，坚持科学发展，坚持改革开放，坚持统筹兼顾，坚持好字优先，坚持以人为本，着力抓好七个方面的工作：

　　(一)打好服务业发展攻坚战，推动服务业上规模上水平，在构建新的支柱产业体系上求突破

　　服务业是我市三大产业中最为薄弱的产业，也是一个发展潜力巨大的产业。要按照“稳一产、强二产、抓三产”的结构调整总体思路，下大决心、花大力气改变我市服务业发展缓慢、比重偏低的现状，力争在未来三年全市服务业增加值占GDP的比重平均每年提高3个以上百分点，由现在的30%提高到2010年的40%左右，使服务业成为我市继煤电产业之后的又一新的支柱产业。

　　要解放思想，开拓创新，着力营造有利于服务业发展的良好氛围。把加快发展服务业作为各级政府的“一把手工程”，切实加强领导，建立健全目标责任体系，加强宏观协调指导，按照市委、市政府出台的发展规划和实施意见，坚定信心，狠抓落实。要坚持以创新理念提升服务业发展层次，以创新举措破解服务业发展难题，以创新机制激活服务业发展的内在动力。加大政策支持力度，制定实施有利于服务业大发展的配套政策，打破市场分割和地区封锁，破除所有制歧视和行业垄断，降低准入门槛，建立公开、平等、规范的准入制度，积极在土地供应、金融支持、税费减免、价格政策上给予最大倾斜。进一步优化发展环境，拓展创业空间，提供创业平台，充分激发人民群众的创业动力和活力，引导千家万户参与服务业发展，实现创业富民。

　　要抓住重点，集中攻坚，加快构建五大产业体系。一是主攻旅游业这个突破口，加快构建旅游文化产业体系。抓好山阴广武边塞军事文化旅游区、朔城区环城墙古城公园、神头海湿地公园、应县释迦文物馆及配套工程、龙首山文化园、右玉古城古堡保护开发、右玉生态旅游景区整体提升、怀仁金沙滩旅游区、平鲁乌龙洞和北固山生态旅游区等十大景点景区建设。二是积极培育现代物流市场，合理建设物流园区，加快构建现代物流产业体系。加速国有企业主辅分离步伐，推动企业内部物流功能剥离，组建专业物流公司，鼓励企业将原材料采购、运输、仓储等供应链管理服务委托给物流企业和物流园区。利用信息化优势，加快设施、设备、运输工具等物流资源的重组整合，鼓励个体私营运输户组建民营运输企业，并逐步向综合服务型物流企业过渡。今年重点抓好市晋冀蒙物流中心、怀仁煤炭集运枢纽、金海洋煤炭物流基地、鲁兴煤炭物流基地、太铁工贸物流中心、怀仁陶瓷交易中心等一批物流基地建设，力争使我市成为山西北部的物流中心和全国现代物流链中的重要节点。三是合理布局，规范经营，全面提升现代机电维修业水平，加快构建现代机电维修产业体系。坚决淘汰以门店式小型维修企业为主的发展方式，围绕工矿机电、机动车辆、家用电器配件供给和维修，着力构建布局合理、经营规范、辐射周边的现代机电维修网络，尽快形成产业规模。重点在市区和每座县城周边建设一座较大规模的工矿机电服务中心、一座重型汽车维修服务城和一座轻型汽车维修服务城，并在市区和县城各建设一条家电维修服务街。四是围绕支柱产业发展，优化科技资源配置，推动科技服务市场化，加快构建科技服务产业体系。以煤炭、电力、陶瓷、化工及农业科技开发为重点，抓好科研基础建设，搞好核心和关键技术开发，培育区域创新体系，整合科技资源，鼓励广大科技人员进入产业竞争前沿，推进科技资源共享平台、科技研究开发平台、科技成果转化平台和科技交流合作平台建设。年内重点扶持和建设平朔高新技术研发中心等10个企业研发中心。五是全面提升酒店餐饮、批发零售和社区服务等传统服务业，加快构建传统服务产业体系。重点抓好市副食品批发市场、豪德光彩贸易广场、市五星级宾馆等一批大型市场和星级酒店建设。

　　各位代表，加快服务业发展，已经成为我市经济发展的一项十分紧迫的任务，无论是从建设资源节约型和环境友好型社会的角度，还是从促进人民群众就业增收、不断提高人民群众生活水平的角度分析，都迫切要求我们必须举全市之力、集全民之智，加快服务业发展。市政府今年将拿出1.5亿元专门用于支持旅游业发展，并决定每年拿出1亿元作为服务业发展基金，拿出2000万元作为引导资金，同时制定了一系列具体的支持鼓励政策。我们坚信，只要坚持不懈地抓下去，一个产业体系健全、经济多元发展的良好格局，很快就将展现在我们面前!

　　(二)改造提升传统工业，发展壮大新兴工业，在建设新型能源和工业基地上求突破

　　要坚定不移地实施“工业强市”战略，紧紧围绕新型能源和工业基地建设目标，继续坚持传统产业新型化和新兴产业规模化方向，坚持不懈推进结构调整，全面提高工业发展水平。

　　着力改造提升传统工业。能源工业是我市的立市之基，发展之本。要继续做好煤电工业这篇大文章，扎实推进煤炭产业的升级改造，加快以节能环保为主的新型电力工业发展，全面提升能源工业素质，增强可持续发展能力，巩固我市能源大市地位。

　　要抓住全省煤炭工业第三战役和全国性煤炭资源整合机遇，鼓励支持大型企业集团整合我市中小型煤矿，基本淘汰年产30万吨以下煤矿，使全市地方煤矿总数压减到100座左右，并形成一批集产运销和加工转化于一体的大型煤炭旗舰企业。着力推进一批大型矿井建设，重点抓好两千万吨的平朔东露矿、千万吨级的麻家梁和梵王寺煤矿、怀仁联顺玺达600万吨矿井整合、金海洋300万吨综采、王坪和小峪煤矿联合开采等大型煤炭项目建设。抓好金海洋、刘家口、国怀精煤、木瓜界、杨涧5座群矿型煤炭洗选设施建设。加快采煤方法升级改造，进一步提高机械化水平。力争到“十一五”末，全市地方煤矿70%以上实现机械化开采，70%以上与大企业大集团合作，煤炭回采率达到70%以上，煤炭洗选率达到70%以上。

　　切实抓好已列入国家电力发展规划的8座电厂建设，集中精力，责任到人，攻坚决战，加快平朔和格瑞特两个已开工电厂的建设，力争其余6个电厂早日开工。同时，积极争取神头一电厂2×60万千瓦关小上大和中煤2×30万千瓦矸石电厂项目获得国家发改委许可。力争到2010年，全市电力装机容量达到800万千瓦以上。

　　精心培育壮大新兴工业。加快现代煤化工、煤机装备制造、新材料、陶瓷、医药保健品等新兴工业的发育成长，加快发展速度，提升产品档次，提高在全市经济构成中的比重，带动工业整体结构优化升级，增强工业经济综合实力和发展后劲。化工业重点抓好中煤公司15万吨多孔硝铵和20万吨浓硝酸扩建、山阴天鹏公司5万吨高效有机肥、右玉全盛公司万吨乳化炸药等项目；新材料工业重点抓好泉鑫1.5万吨单晶莫来石和6万吨干息焦用耐火材料、同煤集团200万吨干法水泥、金海洋130万吨电厂废渣水泥、天磊公司10万吨高岭岩加工等项目；医药业重点抓好同星公司1万吨抗生素、玉龙公司1000吨甲基肼等项目。

　　积极推进循环经济发展。循环经济是有效节约资源、减少污染、提高效益的经济发展新模式，是企业和社会实现双赢的新途径，是工业经济发展的重要方向。要把发展循环经济作为改造传统产业、嫁接新兴产业的主要途径，抓住我省列入全国循环经济试点省的机遇，早动手，早规划，早实施，加紧选择和培养一批试点企业、试点园区，着力抓好平朔、神头电厂、金海洋、刘家口四个循环经济园区建设，突出利用粉煤灰提取二氧化硅和氧化铝项目的工业化推广，力争年内动工兴建。要以发展循环经济来带动节能降耗工作的深入开展。在切实抓好节能降耗各项措施、全面完成节能降耗年度目标任务的同时，把发展循环经济作为节能降耗的持久性、根本性措施来抓，积极推动一批重点能耗企业进行循环式发展，在利用废料、废水、废气、废渣和矿产资源衍生伴生物上谋篇布局、做足文章，努力走出一条能源工业地区循环式、节约化、可持续发展的新路子。

　　要坚持以园区和项目建设推动结构调整。切实抓好市经济开发区、朔城区富甲工业园、怀仁云东经济开发区等十大工业园区的建设，科学界定发展方向和园区定位，加强基础设施建设，增强配套服务能力，加快企业入园步伐，着力打造企业集群发展、集约发展的平台。毫不动摇地实施项目带动战略，切实抓好“三个一百”项目建设工程：确保年内有100个项目开工，100个项目建成投产，100个项目进行前期准备，形成梯度推进格局，保持发展后劲。

　　(三)大力发展特色现代农业，扎实推进新农村建设，在全面提高农业农村发展水平上求突破

　　“三农”问题，直接关系全市近90万农村居住人口的生产生活和生存发展，是最大的民生工程、富民工程。要始终把“三农”工作作为全市经济社会发展的重中之重，以新农村建设为主线，以产业发展为重点，以增加农民收入为目标，努力提高农业农村发展水平。

　　以构筑现代农业产业体系为重点，加快生态畜牧基地建设。按照区域化布局、规模化种植、标准化生产、集约化经营的思路，继续抓好优质高蛋白玉米、高淀粉马铃薯、特色小杂粮和蔬菜、葫麻、甜菜六大基地建设，带动种植业结构调整，使六大基地面积占到农作物总播面积的90%以上，促进优势产业快速发展，确保粮食稳定，蔬菜、葫麻、甜菜总产再创新高。认真实施人畜分离小区、青贮窖、良种繁育体系、机械化奶站“四大工程”建设，新建人畜分离示范小区100个、青贮窖4000个、奶牛配种点100个、小型奶站60个，使全市奶牛业基本实现青贮饲料满足供应、机械化挤奶全面覆盖、规模养殖大面积推开、良种比例大幅度提高，推动畜牧业生产方式重大转变和畜禽品种进一步优化，提高养殖业规模化、科学化、现代化水平。力争全市奶牛存栏量达到16.5万头，生猪和羊饲养量分别达到75万头和250万只，使我市真正成为雁门关生态畜牧经济区建设的排头兵。要按照“科学规划、合理布局、统筹资金、整合项目、选准产业、形成规模、打造精品、示范带动”的要求，抓好城区油坊头、平鲁凤凰城、山阴马营庄、怀仁新家园、应县南河种、右玉威远等六大高标准现代农业综合示范园区建设，力争通过几年的不懈努力，使其成为产业有特色、发展有活力、经济有实力、创新有能力、服务有保障的综合性精品示范区，成为全市现代农业发展的领头雁。

　　以龙头企业建设为重点，提高农业产业化水平。坚持用工业化的理念抓农业，突出抓好“三大方阵”建设：扶持古城、康喜、雁门等15家销售收入超亿元的企业做大做强，向5亿元目标奋进；扶持右玉中大科技和汇源公司、应县雅士利和中粮糖厂等新确定的10家龙头企业尽快投产达效；扶持现销售收入5000万元左右的企业向亿元目标迈进。同时，要围绕产业链条延伸，再着力引进、兴建一批相关龙头企业。通过龙头企业带动，初步形成玉米、马铃薯、小杂粮、蔬菜、葫麻、甜菜、饲草、乳品、肉类和沙棘加工等十条农业产业链，为农业稳步发展、农民持续增收奠定坚实基础。

　　以强化三大支撑为重点，增强农业发展后劲。一是强化基础设施支撑。严格耕地保护，加强农田水利基本建设，提高农业机械化水平，夯实农业发展基础。争取海子湾、东石湖、大梁、耿庄、尚希庄、太平窑二库等六座水库年内全部开工；新建续建灌区节水面积5万亩，完成水保治理60万亩，土地开发整理5万亩，改造中低产田4.5万亩；全市耕播收综合机械化水平比去年提高2个百分点。二是要强化服务支撑。加强农业科技服务、畜禽防疫检疫、农产品市场、“三电合一”信息网络、农产品质量安全检测、农产品质量认证体系建设，增强对现代农业的支撑。建设山西农科院朔州分院，加快平鲁、右玉脱毒种薯繁育中心和右玉小杂粮繁育中心建设；提升35个乡镇畜牧兽医中心站的服务能力，新建50个畜牧兽医分站；建设好市农机大市场和朔城区新世纪、应县南河种、山阴岱岳、怀仁南小寨、平鲁井坪、右玉新城镇等一批农副产品批发市场，建设网上配送销售农产品中心20个；完成农民培训6万人。三是强化政策支撑。不折不扣落实好中央、省和市委市政府出台的一系列强农惠农政策。积极构建“以工促农、以城带乡”的长效机制，探索“以煤补农”、“以企帮村”新途径。建立长效的投资支撑体系，抓住财政收入快速增长的大好时机，大幅度增加对农业农村的投入。整合各类支农资金，集中使用，提高效益。

　　以新农村建设为重点，大力改善农村面貌。按照“分类指导、整体推进”的原则，创新方法，创造特色，扩大试点，攻克弱点，全面提高建设水平。试点村和重点推进村要增加到300个。新增的121个省级重点推进村，年内全面完成一个科技文化活动室、一个卫生计生所、一个健身活动场所、一个便民连锁店、一所标准化学校“五个一”工程建设任务，抓好街巷硬化、村庄绿化、路灯亮化、环境净化和改水、改厕、改灶、改圈“四化四改”工作，同时开展道路通达、信息网络、科技培训、供销服务和文明新风“五进村”活动，形成一批标准较高、发展较快、模式较新的新农村建设典型。大力推进农村净化、绿化、美化工作，促进农村面貌明显改观。着力推进大型沼气设施建设，加快太阳能等清洁能源的推广应用。进一步搞好扶贫开发，实施移民搬迁3000人，整村推进13个村。解决好105个村10万人的饮水安全问题。加快“两区”开发项目建设，力求年内全部竣工。

　　各位代表，农业是一个弱质产业，农民是相对的弱势人群。多少年来，农业和农民为我们的现代化建设与城市繁荣作出了巨大贡献。一部共和国的发展史，就是一部“三农”奉献史。现在，我们有能力、也到了应该补偿农业、补偿农村、补偿农民的时候了。我们一定要带着感情，全心全意地为农业、农村和农民服务，千方百计提高农业农村发展水平，让广大农民跟上现代化发展的步伐，过上更加幸福美满的生活!

　　(四)加快特色城镇化进程，加强生态文明建设，在打造最宜居城市上求突破

　　以迎接建市二十周年为契机，全面推进城镇基础设施建设，加强生态文明建设，加速向碧水蓝天、生活舒适的最宜居城市目标迈进。

　　强力推进中心城市建设。要以大投入、大建设，促进城市规模大扩展、功能大完善、品位大提高。要通过多种渠道，筹资20多亿元，集中抓好20项重点工程，再掀城市建设新高潮。要制定出台创建宜居城市的实施意见和方案，着手城市总体规划的修编和区域控制性详细规划的编制工作，完善城市发展大纲。加快城市道路建设，进一步拉开城市框架。完成西环路建设，实现外环成形；完成开发路南延，与南环路相接；完成大运路市区段拓宽改造，与南环路和大运路市区北段(北环)相连；推进民福街、市府街、古北街、鄯阳街四条大街西延，与西环路相通；开工建设顺义路、友谊街和露北街，完善市区北部路网。通过这些道路建设，使城市主干道基本完善，初步形成100平方公里的城市框架。加快城市公共设施建设，完善城市功能。集中抓好图书馆、博物馆、新闻大楼、文化艺术中心、体育馆、火车站站前广场及候车大厅、公安技侦大楼、海关商务大楼、规划大厅、旧城改造及城中村改造、污水处理厂中水回用、垃圾处理厂等重点工程，力争年内大部分建成使用，向建市20周年献礼。继续推进净化、亮化、绿化“三化”工作，提升城市整体形象。集中抓好城中村垃圾清运、主要街道广告牌匾整治、小街小巷破损道路整修、较大居住小区市场建设等工作，提高净化水平。进一步扩大亮化成果，实现振华街、市府西街、鄯阳街的整体亮化及市区主干街道的楼体亮化。启动国家级园林城市创建工作。市区主干道全部拆墙透绿，展开市区环城绿化带、大运路绿化带和城区古城绿化带“三条绿带”建设，扩大市区主要出入口绿化面积，加强景观建设，力求把城市装扮成干净整洁、流光溢彩、满目翠绿的美丽家园。推进城市人性化管理，提高城市文明程度。

　　加快特色城镇建设步伐。制定完善县域城镇体系规划，加快村镇规划编制。要以旧城改造为契机，优化内部格局，完善基础设施，强化城市功能。择优培育重点中心镇，逐步形成布局合理、结构有序的城镇化体系。要制定优惠政策，吸引各方投入，促进城镇市场发育和人口集聚，不断提高城镇化水平。

　　大力发展交通事业。建设大同至原平高速客运铁路朔州段。建设威海至乌海高速公路朔州段和大同至内蒙高速公路右玉段两条高速公路；完成平鲁至神头电厂、广武至阳方口等运煤通道建设；完成300公里县乡公路改造和400公里农村通达通畅工程；建设村村通油(水泥)路500公里，87%的建制村实现通油(水泥)路，95%的建制村通班车。

　　加强生态文明建设。要象珍惜生命一样珍惜良好生态，象爱护眼睛一样爱护秀美山川。要狠下决心抓治污。以铁的手腕推进污染减排，切实抓好燃煤电厂脱硫除尘改造和污水处理厂建设工程，坚决淘汰落后生产能力。加强项目建设管理，不达环评要求的项目一律不准建设，已经建成经整改后仍不达标的，一律强行关闭。认真实施蓝天碧水工程，彻底取缔市区燃煤茶炉，加快市区垃圾处理厂和医疗废物处理厂建设，全面控制扬尘污染，市区空气质量二级以上天数要保持在300天以上，环境空气质量稳定达到三级标准，争取达到二级。

　　坚持不懈抓造林。集中抓好环城绿化、通道绿化、交通沿线荒山绿化、村庄绿化、城市和厂矿绿化、国家重点生态建设等六大绿化工程，完成造林30万亩，四旁植树500万株，森林覆盖率增加1个百分点。要通过治污增绿，使我市真正成为碧水青山、风景秀丽的宜居城市，成为广大人民群众生存发展的人间乐园!

　　(五)继续深化改革，不断提高开放水平，在增强经济发展活力与动力上求突破

　　全面推进各项改革。继续推进国有集体企业改革。抓好已改制企业的完善提高，下大力气解决好企业改制中的职工养老保险、工资拖欠等遗留问题；下决心推进未改制企业的改革，力争用两年时间基本完成全市国有集体企业改制工作；鼓励引导优秀企业上市，力争两年内有1—2家企业上市融资。深化农村综合改革。健全土地经营权流转市场，加快农村集体林权制度改革，稳步推进水权制度改革。积极推进财政金融体制改革。进一步完善市县乡财政管理体制，探索建立农业保险制度和奶牛风险基金，筹建农村商业银行，尽快启动市金融办公室。继续推进信用担保体系建设，壮大信用担保实力。放手发展民营经济，引导民间资本本地化创业。

　　继续加大招商引资力度。牢固树立“以大开放促进大引进、以大引进促进大发展”的思想，紧紧依靠优良环境、优势产业、优质服务扩大招商引资和引才引智。加强项目库建设，增加项目储备。认真做好项目前期工作，做到资料翔实齐全，谈判预案充分，报批手续完备。加强部门之间的联合协作，做到信息共享、配合协调，提高招商引资的成功率。积极参加各类招商引资活动，组织好第三届中博会和第二届煤博会的参会工作。加强对签约项目的跟踪落实，着重在提高合同兑现率、资金到位率、项目开工率上下功夫，巩固招商引资成果，力争引进非煤非电项目资金90亿元以上。

　　(六)大力实施五大惠民工程，积极推进“和谐朔州”建设，在着力改善民生上求突破

　　各位代表，我们清醒地知道，我们的一切权力都来自于人民，人民群众是我们的衣食之源、权力之本。政府之于人民，一如子女之于父母。一个不为人民群众办事的政府，就是一个忘本的政府。我们一定要坚持把为人民群众谋利益作为政府一切工作的出发点和落脚点，时刻把人民群众的冷暖记心头，特别要从最基层人民群众的安危健康想起，从满足群众最基本的衣食温饱需求做起，自觉运用人民赋予的权力，积极为人民群众办实事、办好事，帮民富、解民忧，保民安、化民怨，用我们的真心真情，尽我们最大的努力，回报朔州广大父老乡亲!

　　致力于学有所教，抓好教育协调发展工程。一是从今年秋季起，全面免除城市义务教育阶段学生学杂费和教科书费，建立健全贫困生资助机制，决不让一个孩子因贫失学。二是进城务工人员子女入学不受户口和地域限制，与市民子女一样平等接受义务教育。三是每个县区在县城新建一所高标准寄宿制中学，逐步满足初中学生到县城上学的需求。四是每个乡镇新建或扩建一所保姆式寄宿制小学，全市共建70所。五是为全市6.4万名农村寄宿制学生每天供应一杯牛奶或一颗鸡蛋。六是开工建设高等师范专科学校，力争明年主体工程竣工，通过国家教育部验收，填补朔州高等教育空白。七是要加快发展职业教育和特殊教育，完善市职业技术学院和聋哑学校配套设施建设，促进教育协调发展。要通过多种措施和办法，让所有孩子都能上得了学、上得起学、上得好学，迈好人生第一步。

　　致力于劳有所得，抓好创业就业工程。坚持以创业带动就业，鼓励自谋职业和自主创业，努力扩大就业机会，大力发展就业吸纳能力强的劳动密集型产业，有效增加就业岗位。切实做好高校毕业生和退伍转业军人就业工作，特别是对零就业家庭，政府要帮助其至少解决一名人员就业，力求做到全市没有一户零就业家庭。

　　致力于住有所居，抓好安康居住工程。人民安居乐业，政府责无旁贷。我市人均住房面积位居全省前列，但其背后掩盖了不少人的住房困难，一些干部群众至今还是无房户。社会的公平与和谐，政府的责任与使命，要求我们必须高度重视，花大力气予以解决。今年要新增廉租住房6万平方米，连同货币补贴，保障总户数达到4430户；新建经济适用住房21万平方米，改造城市棚户27万平方米；完成1000户农村特困群众和300户农村贫困残疾人危房改造。加快市区热源建设，改造供热管网，切实解决好市区供热不足的问题。继续推进住房公积金扩面工作，增加商品房供给，抑制市场房价，规范房地产市场秩序，促进房地产业健康发展，安得广厦千万间，大庇天下寒士俱欢颜!

　　致力于老有所养，抓好社会保障工程。一是要加大保障投入。今年全市财政用于社会保障方面的支出要达到2.56亿元，同比增长53%，其中市级6200万元，增长172%。二是启动山阴、怀仁、应县、右玉65岁以上农村居民生活补贴制度，实现全覆盖，使我市成为全国第一个全面实行农村老人补贴的地级市。三是建设市复退军人精神病院、社会福利院和流浪儿童救助中心。四是每个县区建设一所高标准的中心敬老院。五是进一步完善已出台的12项城乡弱势群体救助制度，扩大救助范围，提高救助标准，努力解决好困难群众的生产生活问题，让公共财政的阳光普照朔州大地，让改革发展的成果惠及每一位朔州人民!

　　致力于病有所医，抓好医疗健康工程。着力改变我市医疗卫生事业发展滞后的状况，努力改善医疗卫生条件。一是进一步完善公共卫生和医疗救助体系，完成市传染病救治中心建设，提高重大疫病防控水平和救治能力。二是高标准完成12所中心乡镇卫生院和200个农村卫生室达标建设。三是使县、乡、村三级医疗卫生机构基础设施达标率达到80%以上。四是城市社区卫生服务机构建设完成规划任务的85%以上，并新建10个社区卫生服务站。五是启动城镇居民基本医疗保险制度，进一步扩大新型农村合作医疗，努力实现城镇医疗保险和新型农村合作医疗两个全覆盖。

　　各位代表，五大惠民工程包含了省市政府要为群众办好的二十件实事的大部分内容。这五大工程、二十件实事，任务繁重，投资巨大，全市需投入4.7亿元，其中市级2亿元。但群众利益无小事，无论任务有多大，工作有多难，资金有多紧，我们都务求保质保量、一件不差、一丝不苟地落实好!

　　同时，要积极加快各项社会事业发展。大力实施科教兴市和人才强市战略，积极推进文化事业发展，完成数字电视平移工程，积极开展群众性体育活动，加强社会主义精神文明建设。着力推进“平安朔州”建设，加强治安防控体系建设，完善信访工作机制，建立健全应急管理体系，为奥运会成功举办创造稳定的社会环境。要加强市场监管，高度重视安全生产，进一步强化人口和计划生育工作，加强国防后备力量建设，大力发展外事、档案、史志、气象、测绘、地震等事业，关心支持老龄、妇女、儿童、残疾人、民族宗教等工作，全面提高和谐社会建设水平。

　　（七）加强政府自身建设，创优发展环境，在建设最宜发展城市上求突破

　　宜发展的核心是软环境，软环境的关键在政府。加强政府自身建设、提高服务水平、创优发展环境，是时代的要求，人民的期盼，更是我们的责任。

　　要解放思想，开拓奋进，努力建设创新型政府。牢固确立不甘落后、勇争一流的宏大志向。坚决破除小富即安、小进则满的自足心理，坚决反对强调客观、顺其自然的消极倾向和得过且过、碌碌无为的庸人作风，大力弘扬“豪爽大气、海纳百川、百折不挠、奋力赶超”的新朔州精神，时时刻刻瞄准先进，以“一争天下无难事”的气魄，不达目的誓不罢休的精神，开拓进取，奋勇向前，努力争创一流业绩。要有敢想敢干、敢闯敢试的创新精神。坚决反对求稳怕乱、怕担风险，不求有功、但求无过，谨小慎微、束手束脚的思想倾向和工作作风，树立无功便是过的观念，打破传统观念和思维定势的束缚，勇于开拓，敢为人先，不断研究新情况，拿出新办法，开创新局面。要营造鼓励创新、宽容失败的良好氛围。切实为敢于创新、勇于负责的同志撑腰壮胆，鼓劲加油。坚持把科学发展观作为解放思想的根本准则，坚决革除一切不符合科学发展观的思想观念、体制机制和政策措施，努力推动全市经济社会又好又快发展。

　　要转变职能，尽职守责，努力建设服务型政府。加快由“权力政府”向“服务政府”转变，由“全能政府”向“有限政府”转变，把“服务”作为政府职能的核心，着力解决好政府工作越位、缺位、错位的问题。要竭尽全力为企业提供服务与扶持，竭尽全力为企业排除干扰、创造良好外围环境，竭尽全力维护良好市场秩序、促进公平竞争，由企业和市场的“婆婆”变为“保姆”，由“主宰者”变为“服务生”、“守护神”。要更加注重社会管理和公共服务，竭尽全力促进和谐社会建设，努力提供更多更好的公共服务产品。进一步深化行政审批制度改革，减少审批事项，优化工作流程，精简办事程序，提高办事效率。积极推进政府决策的科学化、民主化进程，建立和完善重大问题集体决策制度、专家咨询制度、社会公示和听证制度、决策责任追究制度，使我们的决策更加符合朔州实际，更加符合人民群众的意志。

　　要依法行政，规范行为，努力建设民主法治政府。要依法治“官”，依法治“权”，依法治事。认真贯彻《行政许可法》和国务院《全面推进依法行政实施纲要》，严格依法行政，依法办事。加快建立权责明确、行为规范、监督有效、保障有力的行政执法体制，减少执法层次，规范执法行为，着力解决多头执法、重复执法和交叉执法问题。推行行政执法责任制和错案追究制，坚决查处徇私枉法、执法犯法、欺压百姓等违法行为。建立健全有效的行政监督体制和机制。各级政府要自觉接受同级人大及其常委会的监督，认真执行人大决议，定期向人大报告工作；积极接受人民政协的民主监督，认真听取各民主党派、无党派人士和各人民团体的意见；加强基层民主建设，认真贯彻国家《政府信息公开条例》，继续推进“三务”公开；拓宽新闻监督、公众监督渠道，做到有权必有责、用权受监督、侵权要赔偿，着力解决权力与利益挂钩、与责任脱钩的问题，确保人民赋予的权力真正服务于人民。

　　要严肃政令，改进作风，努力建设勤政高效政府。政令通则人气和，人气和才能事业兴。要加强对县区政府和所属部门的管理约束，做到有令必行，有禁必止，政令畅通，杜绝推诿扯皮，防止政府权力部门化、部门权力个人化。建立严格的目标责任制，每项重大工作都要目标明确，责任到人，措施具体，做到人人有事干，事事有人干。要强化督促检查，以事查人，以人查事，奖优罚劣，追责问责，严格整治“当官不负责任、只吃俸禄不干实事”的不作为问题，抓出谋事干事的激情。要改进作风，认真贯彻市委关于改进干部作风的十项制度，大兴调查研究、求真务实之风，大兴勤俭节约、艰苦创业之风，大兴团结协作、合力攻坚之风，真正在实干和落实上下真功、用苦功。牢固树立环境就是资源、环境就是效益、环境就是发展资本和发展空间的理念，坚决整治“门难进、脸难看、事难办”的衙门作风和“不给好处不办事、给了好处乱办事”的乱作为问题，还社会以清风正气，给人民以公道方便，真正实现“政府创造环境、人民创造财富”!

　　要强化监督，严格自律，努力建设清正廉洁政府。加强党风廉政建设，建立健全教育、制度、监督并重的惩治和预防腐败体系。认真贯彻中纪委《严格禁止利用职务上的便利谋取不正当利益的若干规定》，继续加强建设工程招标、经营性土地出让、产权交易、资源使用、重大项目投资、政府采购等制度建设，从源头上预防腐败现象滋生。抓好领导干部经济责任审计。认真开展煤炭领域党风廉政建设和反腐败专项治理，深入推进治理商业贿赂工作。认真解决严重损害群众利益的突出问题，纠正部门和行业不正之风，坚决查办违法违纪案件，严惩腐败分子。加强廉政文化建设和廉政教育，使每个干部，特别是领导干部，都能做到以谨慎之心对待权力，以淡泊之心对待名利，以警惕之心对待诱惑，谨言慎行，警钟长鸣，干干净净为国家和人民干事。

　　各位代表，朔州是一个充满生机与活力的地方，朔州的明天必将是无比光明灿烂的!现在，我们已经站到了一个新的历史起点上，让我们在中共朔州市委的坚强领导下，高举中国特色社会主义伟大旗帜，以邓小平理论和“三个代表”重要思想为指导，全面落实科学发展观，紧紧团结和依靠全市人民，解放思想，科学发展，奋力赶超，为把我市建设成为塞外最宜居最宜发展城市而努力奋斗！
